# *Bt* rice does not disrupt the host-searching behavior of the parasitoid *Cotesia chilonis*

**DOI:** 10.1038/srep15295

**Published:** 2015-10-15

**Authors:** Qingsong Liu, Jörg Romeis, Huilin Yu, Yongjun Zhang, Yunhe Li, Yufa Peng

**Affiliations:** 1 State Key Laboratory for Biology of Plant Diseases and Insect Pests, Institute of Plant Protection, Chinese Academy of Agricultural Sciences, Beijing, China; 2 Agroscope, Institute for Sustainability Sciences ISS, Zurich, Switzerland

## Abstract

We determined whether plant volatiles help explain why *Cotesia chilonis* (a parasitoid of the target pest *Chilo suppressalis*) is less abundant in *Bt* than in non-*Bt* rice fields. Olfactometer studies revealed that *C. chilonis* females responded similarly to undamaged *Bt* and non-*Bt* rice plants. Parasitoids preferred rice plants damaged by 3^rd^-instar larvae of *C. suppressalis,* but did not differentiate between caterpillar-infested *Bt* and non-*Bt* plants. According to GC-MS analyses of rice plant volatiles, undamaged *Bt* and non-*Bt* rice plants emitted the same number of volatile compounds and there were no significant differences in the quantity of each volatile compound between the treatments. When plants were infested with and damaged by *C. suppressalis* larvae, both *Bt* and non-*Bt* rice plants emitted higher numbers and larger amounts of volatile compounds than undamaged plants, but there were no significant differences between *Bt* and non-*Bt* plants. These results demonstrate that the volatile-mediated interactions of rice plants with the parasitoid *C. chilonis* were not disrupted by the genetic engineering of the plants. We infer that parasitoid numbers are lower in *Bt* than in non-*Bt* fields because damage and volatile induction by *C. suppressalis* larvae are greatly reduced in *Bt* fields.

Genetically engineered (GE) crops have been widely adopted, with 181 million hectares of GE crops grown globally in 2014^1^. Nearly half of these crops were modified by inserting *cry* genes from *Bacillus thuringiensis* (*Bt*). These genes encode insecticidal Cry proteins that can suppress lepidopteran and coleopteran pests. Production of insect-resistant GE (IRGE) plants has increased farmer incomes and decreased the use of broad-spectrum chemical insecticides that endanger human and ecosystem health^1^,^2^,^3^,^4^.

Despite these benefits, the potential environmental risks associated with the planting of IRGE crops have received substantial attention, with a particular focus on the potential effects on valued nontarget organisms. Among nontarget organisms, natural enemies of pest arthropods are of particular interest because they help regulate herbivore populations and thus contribute to sustainable agro-ecosystems^5^. Many field studies have assessed the potential effects of *Bt* crops on the abundance of nontarget arthropods, and meta-analyses indicate that nontarget species are generally more abundant in *Bt* cotton and *Bt* maize fields than in non-transgenic fields managed with insecticides^6^,^7^,^8^,^9^. When *Bt* fields are compared with insecticide-free control fields, however, nontarget taxa – such as specialist parasitoids of target pests – are less abundant in *Bt* fields^6^,^7^,^8^,^9^. The reduced abundance of parasitoid wasp species of the target pests in *Bt* crop fields is associated with the lack of their hosts and does not appear to be caused by toxicity of the *Bt* Cry proteins^7^,^8^,^9^.

In searching for their hosts, parasitoids often rely on the chemical communication between the parasitoids, their hosts, and/or the host plant. More specifically, parasitoids can use herbivore-induced plant volatiles (HIPVs) to locate their hosts^10^,^11^,^12^,^13^. The lower abundance of parasitoid species in *Bt* than in non-*Bt* crops might result from differences in plant volatiles as a consequence of two potential mechanisms: i) the insertion of foreign insecticidal genes may change the production of plant volatiles due to pleiotropic effects, epistasis, or insertional mutagenesis; and ii) compared to *Bt* plants, non-*Bt* plants will be more heavily damaged by target pests and thus release greater amounts of plant volatiles or some new volatiles, making them more attractive to the parasitoid wasps. A few studies have investigated the potential effects of *Bt* plants on parasitoid behavior as mediated by plant volatiles. Most of these studies have reported that undamaged *Bt* plants and the corresponding undamaged non-*Bt* control plants emitted similar types and quantities of volatiles and were similarly attractive to parasitoid species; when infested with and damaged by caterpillars, however, the non-*Bt* plants receive more damage than the *Bt* plants and consequently release qualitatively and quantitatively different volatile profiles, resulting in higher attractiveness to parasitoid wasps^14^,^15^,^16^,^17^,^18^,^19^. Although Yan *et al.*^20^ reported that the expression of a foreign gene in *Bt* cotton plants affected their volatile profiles, the authors did not determine whether this difference affected insect behavior.

For controlling lepidopteran rice pests, such as the striped stem borer, *Chilo suppressalis* (Lepidoptera: Crambidae), the yellow stem borer, *Scirpophaga incertulas* (Lepidoptera: Pyralidae), and the rice leaf roller, *Cnaphalocrocis medinalis* (Lepidoptera: Pyralidae), various IRGE rice cultivars have been developed in China; these IRGE rice cultivars express the *Bt* genes *cry1Ab, cry1Ac, cry1Aa, cry1Ab/Ac, cry2Aa*, or *cry1C*^21^,^22^. The IRGE rice lines exhibit high levels of resistance against the target pests, and laboratory and glasshouse studies have verified that the Cry proteins produced by *Bt* rice plants have no negative effects on nontarget species outside the order of Lepidoptera^22^,^23^,^24^,^25^,^26^,^27^,^28^. Field studies confirmed that the planting of *Bt* rice did not significantly affect populations of non-target arthropods, with the exception of parasitoid wasps that attack the target pests^29^,^30^,^31^. No data have been reported regarding the potential effect of *Bt* rice plants on the host-searching behavior of parasitoid wasps.

The transgenic rice line T1C-19 expresses a synthesized *cry1C* gene and has a high level of resistance to lepidopteran insect pests including stem borers and leaf folders^24^,^32^. T1C-19 thus has the potential to be commercialized in China, and was used in the current study to investigate the effects of *Bt* genetic engineering on the host-searching behavior of parasitoid wasps. Our study system consisted of the *Bt* rice line T1C-19, its non-transformed parental rice line, the target pest *C. suppressalis*, and its parasitoid *Cotesia chilonis* (Hymenoptera: Braconidae). We performed a series of olfactory experiments to evaluate the attractiveness of volatiles from undamaged or caterpillar-damaged plants of both rice lines to the parasitoid. In addition, the volatiles from undamaged and damaged plants were characterized in order to determine whether there are qualitative or quantitative differences in the volatile bouquet produced by the *Bt* and non-*Bt* plants.

## Results

### Response of *C. chilonis* to rice plants damaged by 3^rd^-instar *C. suppressalis* larvae

The mean body weight increase of 3^rd^-instar *C. suppressalis* larvae was 0.91 ± 0.14 mg when fed non-*Bt* rice stems, and was 0.75 ± 0.12 mg when fed *Bt* rice stems for 24 h. However, the difference detected between the two treatments was not significant (*t *= −0.851, df = 64, P = 0.398).

**Figure 1:**
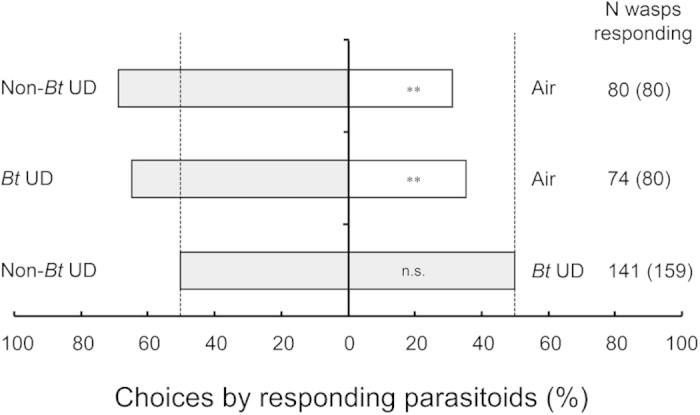
Responses of *Cotesia chilonis* to pairs of odor sources in dual-choice tests using a Y-tube olfactometer. Odor sources: *Bt* UD: undamaged *Bt* rice plants, non-*Bt* UD: undamaged non-*Bt* rice plants, Air: clean air. Asterisks indicate a significant difference within a choice test: **P < 0.01; n.s. indicates a non-significant difference (*P *> 0.05) (χ^2^ test). The bars indicate the percentages of parasitoids that selected either odor source. Gray shading indicates herbivore-undamaged plants. The numbers indicate the number of parasitoids that made a choice, and the numbers in parentheses indicate the total number of parasitoids tested.

Females of the parasitoid *C. chilonis* were more strongly attracted to the odors of undamaged (UD) *Bt* or non-*Bt* rice plants than to clean air (non-*Bt* UD vs. Air, χ^2 ^= 10.60, *P* = 0.001; *Bt* UD vs. Air, χ^2 ^= 7.2, *P *= 0.007) (Fig. 1). The attraction of the parasitoid to undamaged *Bt* vs. undamaged non-*Bt* rice plants did not differ significantly (*Bt* UD vs. non-*Bt* UD, χ^2 ^= 0.007, *P *= 0.933) (Fig. 1).

**Figure 2:**
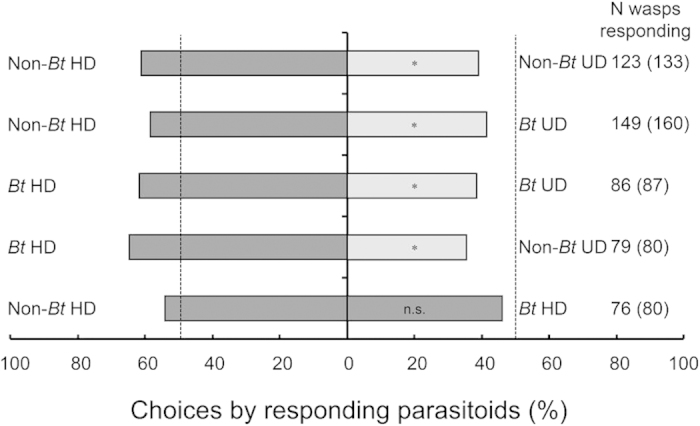
Response of *Cotesia chilonis* to pairs of odor sources in dual-choice tests using a Y-tube olfactometer. Odor sources: *Bt* UD: undamaged *Bt* rice plants, non-*Bt* UD: undamaged non-*Bt* rice plants, *Bt* HD: *Bt* rice plants damaged by 3^rd^-instar *C. suppressalis* larvae, and non-*Bt* HD: non-*Bt* rice plants damaged by 3^rd^-instar *C. suppressalis* larvae. Asterisks indicate a significant difference within a choice test: *P < 0.05; n.s. indicates a non-significant difference (*P *> 0.05) (χ^2^ test). The bars indicate the percentages of parasitoids that selected either odor source. Dark shading indicates herbivore-damaged plants and gray shading indicates herbivore-undamaged plants. The numbers indicate the number of parasitoids that made a choice, and the numbers in parentheses indicate the total number of parasitoids tested.

*C. chilonis* females were more attracted to rice plants damaged by 3^rd^-instar *C. suppressalis* larvae (HD) than to undamaged plants regardless of plant genetics (either *Bt*-transgenic or non-transformed) (non-*Bt* HD vs. non-*Bt* UD, χ^2 ^= 5.93, *P *= 0.015; non-*Bt* HD vs. *Bt* UD, χ^2^ = 4.20, *P *= 0.041; *Bt* HD vs. *Bt* UD, χ^2 ^= 4.65, *P *= 0.031; *Bt* HD vs. non-*Bt* UD, χ^2 ^= 6.70, *P *= 0.001) (Fig. 2). When given a choice between herbivore-damaged *Bt* and non-*Bt* plants (*Bt* HD vs. non-*Bt* HD), the parasitoids were equally distributed between the two arms of the olfactometer (χ^2^ = 0.47, *P *= 0.491) (Fig. 2).

### Response of *C. chilonis* to rice plants damaged by 1^st^- or 3^rd^-instar *C. suppressalis* larvae

**Figure 3:**
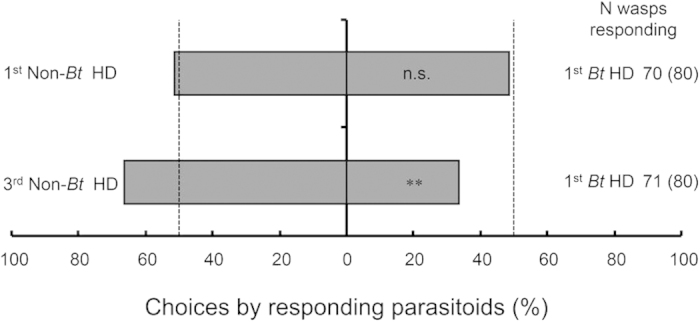
Response of *Cotesia chilonis* to pairs of odor sources in dual-choice tests using a Y-tube olfactometer. Odor sources: 1^st^ non-*Bt* HD: non-*Bt* rice plants infested with 30 1^st^-instar larvae of *C. suppressalis*, 1^st^ *Bt* HD: *Bt* rice plants infested with 30 1^st^-instar larvae of *C. suppressalis*; and 3^rd^ non-*Bt* HD: non-*Bt* rice plants infested with 10 3^rd-^instar larvae of *C. suppressalis*. Asterisks indicate a significant difference within a choice test: **P < 0.01; n.s. indicates a non-significant difference (*P *> 0.05) (χ^2^ test). The bars indicate the percentages of parasitoids that selected either odor source. The numbers indicate the number of parasitoids that made a choice, and the numbers in parentheses indicate the total number of parasitoids tested.

*C. chilonis* females did not exhibit a significantly different preference for *Bt* or non-*Bt* rice plants that were damaged by 30 1^st^-instar *C. suppressalis* larvae (1^st^ non-*Bt* HD vs. 1^st^ *Bt* HD; χ^2 ^= 0.057, *P *= 0.811). In contrast, non-*Bt* plants infested with 3^rd^-instar larvae were significantly more attractive to *C. chilonis* females than were *Bt* rice plants infested with 1^st^-instar *C. suppressalis* larvae (3^rd^ non-*Bt* HD vs. 1^st^ *Bt* HD; χ^2^ = 7.451, *P *= 0.006) (Fig. 3).

### Volatile analyses

**Figure 4:**
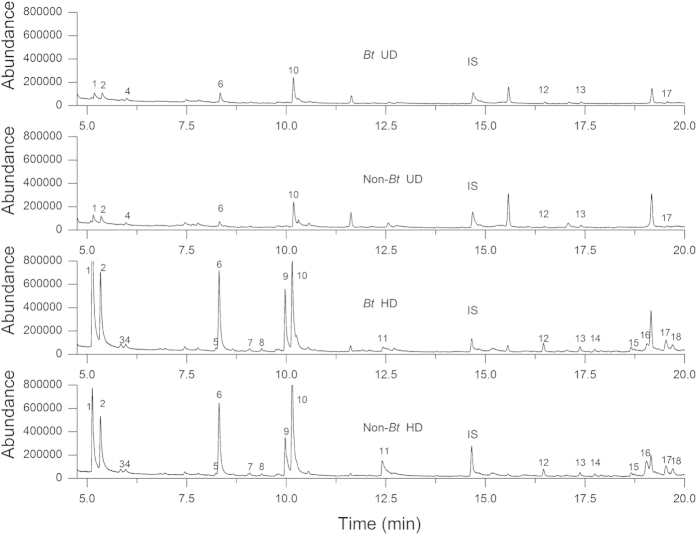
Chromatographic profiles of volatiles obtained from headspace collections from rice plants. Undamaged *Bt* rice plants (*Bt* UD), undamaged non-*Bt* rice plants (non-*Bt* UD), herbivore *C. suppressalis*-damaged *Bt* rice plants (*Bt* HD), and herbivore-damaged non-Bt line (non-*Bt* HD). Peaks: 1, 2-heptanone; 2, 2-heptanol; 3, unknown 1; 4, α-piene; 5, unknown 2; 6, D-limonene; 7, unknown 3; 8, unknown 4; 9, 2-nonanone; 10, linalool; 11, methyl salicylate; 12, copaene; 13, β-caryophyllene; 14, α-bergamotene; 15, germacrene D; 16, 2-tridecanone; 17, δ-selinene; 18, α-cedrene. The added reference compound was 1-tridinene (IS).

There were great differences in the composition and quantities of volatiles released by the herbivore-damaged and undamaged *Bt* and non-*Bt* rice plants (one-way ANOVA: *F *= 21.88, d.f. = 3, *P *< 0.001 for volatile quantity) (Table 1 and Fig. 4). Pair-wise comparisons showed that undamaged *Bt* and undamaged non-*Bt* rice plants emitted the same numbers and similar amounts of volatiles (*P *=  0.374) (Table 1). When damaged by caterpillars, both *Bt* and non-*Bt* rice plants released significantly higher amounts of volatiles and several new compounds (unknown 1, unknown 2, unknown 4, 2-nonanone, methyl salicylate, α-bergamotene, germacrene D, 2-tridecanone, and α-cedrene). The total amounts of volatiles released by herbivore-damaged rice plants were 4.6- and 4.8-times higher for *Bt* and non-*Bt* rice plants, respectively, than for undamaged rice plants (*Bt* HD vs. *Bt* UD, *P *< 0.001; non-*Bt* HD vs. non-*Bt* UD, *P *< 0.001) (Table 1). The total amount of volatiles emitted did not significantly differ between damaged *Bt* plants and damaged non-*Bt* plants (*P *= 0.954); the mean quantities of individual volatiles also did not significantly differ between damaged *Bt* plants and damaged non-*Bt* plants (all *P *> 0.05) (Table 1).

## Discussion

The results from our olfactometer tests showed that female *C. chilonis* parasitoids, when given a choice between clear air and odor emitted by undamaged rice plants, strongly responded to the undamaged rice plants whether they were *Bt* or non-*Bt*. This finding is consistent with our GC-MS analyses: undamaged *Bt* and non-*Bt* rice plants released similar types of volatile compounds at similar concentrations. The bouquet that we identified is comparable to that reported in previous studies for other conventional rice lines^33^,^34^,^35^. Our results suggest that genetic engineering of the *Bt* rice line T1C-19 has not altered the secondary metabolism of rice with respect to volatile synthesis/emissions. Similar results have been reported by Sun *et al.*^34^, who found that *Bt*-transgenic rice plants expressing the *cry2A* gene did not emit a different volatile spectrum than the non-transformed control at the seedling, booting, and tillering stages. A number of previous studies have reported that parasitic wasps did not differentiate between healthy undamaged *Bt* and non-*Bt* plants when given a choice. For example, Moraes *et al.*^15^ reported that the egg parasitoid *Trichogramma pretiosum* (Hymenoptera: Trichogrammatidae) showed an equal preference for undamaged *Bt* and non-*Bt* cotton plants. Similarly, the larval parasitoids *Microplitis mediator* (Hymenoptera: Braconidae)^36^ were equally attracted to undamaged *Bt* and non-*Bt* maize. In addition, the adult stages of some phytophagous insects, which also use plant volatiles to locate their host plants, did not show any preference for *Bt* or non-*Bt* plant lines^34^,^37^,^38^,^39^,^40^,^41^,^42^,^43^,^44^,^45^,^46^. In contrast, Yan *et al.*^20^ reported that higher concentrations of α-pinene, β-pinene, and an unknown minor compound were emitted by *Bt* cotton than by non-*Bt* cotton. However, Yan *et al.*^20^ did not determine whether this difference was caused by the insertion of the *cry* gene or whether this difference affected the searching behavior of phytophagous insects or parasitoid wasps.

It is well known that plant volatiles induced by herbivore damage can serve as host-searching cues for natural enemies^8^,^10^,^11^,^13^,^17^,^19^. More specifically, a previous study confirmed that *C. chilonis* females strongly prefered rice plants damaged by *C. suppressalis* larvae to undamaged rice plants^47^. Our results also demonstrate that *C. chilonis* females were more attracted to rice plants damaged by 3^rd^-instar *C. suppressalis* larvae than to healthy rice plants. The GC-MS analyses confirmed that damaged *Bt* and non-*Bt* rice plants emitted significantly larger amounts of volatiles than healthy rice plants. In addition, the damaged plants released several new chemicals, including 2-nonanone, methyl salicylate, α-bergamotene, germacrene D, 2-tridecanone, α-cedrene, and two unknown compounds.

Because *C. suppressalis* is a target pest of *Bt* rice, and because its larvae are expected to cause less damage to *Bt* plants than to non-*Bt* plants, it is reasonable to hypothesize differences in the volatile bouquet and in parasitoid behavior between caterpillar-infested *Bt* and non-*Bt* rice plants. These differences, however, were not evident in the current study. This may be explained by the fact that both *Bt* and non-*Bt* rice plants can be infested with 3^rd^-instar *C. suppressalis* and that such larvae cause comparable damage to *Bt* and non-*Bt* rice plants during a 24-hour duration of feeding. This interpretation was confirmed by our observation that the mean weight increase of 3^rd^-instar *C. suppressalis* larvae fed non-*Bt* rice plants was only a little higher than those fed *Bt* rice plants, a non-significant difference. These results suggest that the planting of *Bt* rice plants will not affect the host-searching behavior of the larval parasitoid *C. chilonis*. Nevertheless, higher densities of parasitoids (including *C. chilonis*) have been recorded in non-*Bt* rice fields than in *Bt* rice fields^29^,^48^,^49^. This could be explained by the fact that *Bt* rice plants are much less damaged in the field than in our study^24^. In contrast to our experiments, where three rice plants were infested with six 3^rd^-instar *C. suppressalis*, plants in the field would be infested by neonates that would be effectively killed by the *Bt* toxin before they cause significant damage. Consequently, parasitoids in the field are likely to be more attracted to or arrested by non-*Bt* rice plants than by *Bt*-rice plants, because the non-*Bt* plants would support much higher densities of lepidopteran larvae as hosts and will probably also release higher amounts of volatiles in response to herbivore damage. Our olfactometer tests with 1^st^- and 3^rd^-instar larvae of *C. suppressalis* could better reflect the field condition. The results showed that the parasitoids did not distinguish between *Bt* and non-*Bt* rice plants that were damaged by 1^st^-instar *C. suppressalis*, although it was expected that the neonates might cause higher damage on non-*Bt* rice plants compared to *Bt* plants. These results may suggest that the relatively little damage caused by neonates of *C. suppressalis* within 24 h on either *Bt* or non-*Bt* plants is not enough to affect the host-searching behavior of the parasitoid *C. chilonis*. However, female *C. chilonis* were significantly more attracted to the odors emitted by non-*Bt* rice plants damaged by 3^rd^-instar *C. suppressalis* than to *Bt* rice plants damaged by 1^st^-instar *C. suppressalis*, a scenario that is likely to occur under field conditions. These results further explained the ecological phenomenon that higher densities of *C. chilonis* are observed in non-*Bt* rice fields than in *Bt* rice fields.

Our results agree with those of earlier studies with *Bt* cotton. Moraes *et al.*^15^ reported that 2^nd^-instar *Spodoptera frugiperda* (Lepidoptera: Noctuidae) larvae caused damage to both *Bt* and non-*Bt* cotton, and that damaged plants emitted a higher quantity of volatiles than undamaged plants. Although the parasitoid *T. pretiosum* was significantly attracted to damaged cotton plants, it did not distinguish between *Bt* and non-*Bt* plants. Several studies have documented that the greater damage caused by the target pests on non-*Bt* plants than on *Bt* plants caused a larger induction of volatiles or plant defense compounds in the non-*Bt* plants; this difference has been reported for cotton^50^,^51^, maize^16^, and oilseed rape^14^,^18^,^19^,^52^. It was also reported that herbivore-damaged non-*Bt* oilseed rape plants were more attractive to the parasitoids *Cotesia plutellae* (Hymenoptera: Braconidae)^18^,^19^ and *C. vestalis* (Hymenoptera: Braconidae)^52^ than were *Bt*-transgenic oilseed rape plants. As expected, when larvae of a *Bt*-resistant *Plutella xylostella* (Lepidoptera: Plutellidae) strain were used in such studies, they caused equal feeding damage on *Bt* and non-*Bt* oilseed rape, and the parasitoid of *P. xylostella* was equally attracted to damaged *Bt* and non-*Bt* oilseed rape plants^18^,^19^. In some studies, although herbivore-damaged non-*Bt* plants released significantly higher amounts of volatiles than the less-damaged *Bt* plants, parasitoid wasps did not differentiate between *Bt* and non-*Bt* plants^16^. This phenomenon was explained by the fact that the ratio of different functional chemical compounds emitted by *Bt* and non-*Bt* plants was not altered.

Our study is the first to elaborate the mechanism by which the populations of parasitoid wasps of the target pest are affected by volatiles released from *Bt* and non-*Bt* rice. The results suggest that the volatile-mediated interactions between rice plants and a parasitoid of a lepidopteran rice pest are not disrupted by the genetic engineering of the rice plant.

## Materials and Methods

### Plants

The transgenic *Bt* rice line, T1C-19, and its corresponding non-transformed near-isoline, Minghui63 (MH63), were used in this study. T1C-19 plants express a synthesized cry1C* gene driven by the maize ubiquitin promoter; the gene encodes the Cry1C protein that targets lepidopteran rice pests^32^. MH63 is an elite *indica* restorer line for cytoplasmic male sterility in China. The rice seeds were provided by Prof. Yongjun Lin (Huazhong Agricultural University, Wuhan, China).

Pre-germinated seeds of both lines were sown simultaneously in a greenhouse, and the seedlings were transplanted after three weeks into individual plastic pots (630 cm^3^) containing a mixture of peat and vermiculite (3:1). Plants were watered daily and supplied with nitrogenous fertilizer every 3 d. All plants were maintained in a glasshouse at 27 ± 3 °C with 65 ± 10% RH and a 16 L : 8 D photoperiod. Four weeks after transplanting, the plants were at the tillering stage and were used for the experiments. A previous study had revealed that at this growth stage, the target pest *C. suppressalis* is almost 100% controlled, i.e., *Bt* rice plants at this growth stage experience no or extremely low damage from *C. suppressalis* feeding^24^.

### Insects

The *C. suppressalis* specimens used in this study were obtained from a laboratory colony that had been reared on an artificial diet^53^. The *C. chilonis* wasps used in this study were provided by Prof. Gongyin Ye (Zhejiang University, Hangzhou, China) and were obtained from a laboratory colony that had been reared on the larvae of *C. suppressalis*. The parasitoid colony has been kept in the laboratory for nearly 20 generations, while field-collected parasitoids were introduced yearly to the colony to introduce fresh genetic material. Each *C. suppressalis* larva (4^th^–5^th^ instar) was exposed to a mated female wasp in a glass tube (2.4 cm diameter, 7 cm height) for 24 h to ensure that the larva had been parasitized. Parasitized larvae then were provided with sufficient artificial diet until they yielded parasitoid cocoons or pupated. The cocoons were collected and transferred to clean Petri dishes (9 cm diameter) until adult emergence. If not used immediately, the cocoons were stored at 15 ± 1 °C and 70 ± 5% RH for up to 2 weeks. Newly emerged adult wasps were supplied with a honey : water solution (20%, v-v) as a food source and were allowed to mate freely for >24 h. Mated female wasps (2 to 3 days old) were used in the bioassays. Female wasps were naïve, i.e., they had not previously contacted rice plants or hosts. Both *C. suppressalis* and *C. chilonis* were kept in environmental chambers (RXZ, 380 L, Ningbo Jiangnan Instrument Factory, Zhejiang, China) at 27 ± 1 °C with 70 ± 5% RH and a 16 L:8 D photoperiod.

### Chemicals

Super Q (80–100 mesh) was purchased from Alltech (Deerfield, IL, USA). Methylene chloride (≥99.8% purity) and 2-nonanone (99% purity), were purchased from Aladdin Reagent Co., Ltd. (Shanghai, China). Linalool (97% purity), 2-heptanol (98% purity), 2-heptanone (99% purity), methyl salicylate (≥99% purity), β-caryophyllene (≥98.5% purity), α-pinene (≥99% purity), D-limonene (≥99% purity), and 1-tridecene (96% purity) were purchased from Sigma-Aldrich (St. Louis, MO, USA).

### Response of *C. chilonis* to rice plants damaged by 3^rd^-instar *C. suppressalis* larvae

#### Plant treatments

The potted rice plants were washed with running water to remove soil and silt. Three plants were placed in 50-ml conical flasks filled with distilled water, and the plants and flasks were wrapped with aluminum foil. Two plant treatments were compared for both *Bt* and non-*Bt* rice plants: (i) undamaged plants (UD), and (ii) herbivore-damaged plants (HD). The HD plants had been infested with six early 3^rd^-instar *C. suppressalis* larvae for 24 h. The larvae had previously been starved for 2 h. Visual observations indicated that 3^rd^-instar larvae of *C. suppressalis* could cause similar damage on *Bt* and non-*Bt* rice plants within 24 h. Subsequently, undamaged or herbivore-damaged plants were enclosed in a frosted glass bottle (3142 cm^3^); a port at the bottom was used for the inlet of air, and a port at the top was used for the outlet of air. Plants in these bottles were used for the olfactometer bioassays and for the collection of volatiles as described in the following sections. During the olfactometer bioassay and the collection of volatiles, *C. suppressalis* larvae were left on plants. All experiments were conducted at 27 ± 2 °C and 40% RH. The lighting level in the frosted glass bottle was 3000 ± 150 Lux.

### Damage on rice plants by 3^rd^-instar larvae of C. suppressalis

To quantitatively measure the degree of damage to *Bt* and non-*Bt* rice plants by 3^rd^-instar larvae of *C. suppressalis*, a bioassay was conducted in which *Bt* and non-*Bt* rice stems were provided to the larvae for 24 h. A thin, moist layer of absorbent cotton was laid on the bottom of a Petri dish (6 cm diameter) and a moistened filter paper (5.5 cm diameter) was placed on the cotton layer. Subsequently two 4–5-cm segments that were cut from the middle part of the main rice plant stems were placed onto the filter paper. One end of the stem segment was covered with saturated cotton wool to keep the rice stems fresh. After being weighed on an electronic balance (Mettler Toledo ME204, Zurich, Switzerland; 220 g full scale, d = 0.0001 g), the 3^rd^-instar larvae of *C. suppressalis* were individually transferred to each Petri dish. The Petri dish then was sealed with Parafilm. A total of 29 and 37 larvae were tested for *Bt* and non-*Bt* rice, respectively. The insects were kept in an environmental chamber (RXZ, 380 L, Ningbo Jiangnan Instrument Factory, Zhejiang, China) under a 16 L:8 D photoperiod at 27 ± 1 °C, 70 ± 5% RH. After 24 h feeding, *C. suppressalis* larvae were weighed again, and the weight increase of each insect was calculated.

#### Olfactometer bioassays

The rice plants enclosed in glass bottles as described in the previous section were used as odor sources. Dual-choice (Y-tube) olfactometers (16-cm stem; 16-cm arms at 75° angle; 2 cm internal diameter) were used to investigate the behavioral responses of naïve *C. chilonis* females to one odor source vs. a blank (clean air) or pairs of different odor sources. The experiments were conducted in a chamber (110 × 80 × 70 cm) formed with iron sheets and equipped with four 16-W cool white fluorescent lights at the top, and the light was filtered by an acrylic plate to ensure an even distribution of light (1650 ± 10 Lux). In addition, a light meter (Shenzhen Huachang Technology Co., Ltd., China) was used to monitor the light distribution. An airstream was generated by a membrane pump (Beijing Institute of Labour Instruments, Beijing, China). Air was filtered through activated charcoal, humidified by passage through a jar containing distilled water, and passed through the odor source before entering the olfactometer. The air-flow through the two olfactometer arms was monitored by two separate airflow meters (Changzhou Kede Thermo-technical Instrument Co., Ltd., Changzhou, China) at 250 ml/min. All parts of the system were connected by 0.6-cm-diameter Teflon tubes.

Individual *C. chilonis* females were introduced into the Y-tube at the base of the stem, and the position of the wasp was observed for 5 min. A ‘first choice’ was recorded when the wasps moved over a threshold line at the middle of either arm for at least 5 s. Wasps that had not made a choice during the observation time were recorded as ‘no choice’, and their data were not included in the statistical analyses. Each wasp was tested only once. A total of 80–160 females were tested for each treatment. All tests were conducted between 9:00 and 18:00 h in a climate-controlled laboratory room (27 ± 2 °C, 40% RH). The position of the arms of the Y-tube were reversed after two tests (replicates) to avoid position bias, and the Y-tube was replaced with a clean one after it was used to test four wasps. To compensate for any unforeseen asymmetry in the bioassays, the odour sources were swapped after 10 individuals were tested and were replaced by a new set of plants after they were used to test 20 wasps. All glassware and connected Teflon tubes were washed with detergent (5%, v/v) followed by alcohol (90%, v/v) and then were rinsed with distilled water. The female parasitoids were exposed to the following eight pairs of odor sources: (i) *Bt* UD vs. clean air, (ii) non-*Bt* UD vs. clean air, (iii) *Bt* UD vs. non-*Bt* UD, (iv) *Bt* HD vs. *Bt* UD, (v) *Bt* HD vs. non-*Bt* UD, (vi) non-*Bt* HD vs. non-*Bt* UD, (vii) non-*Bt* HD vs. *Bt* UD, and (viii) *Bt* HD vs. non-*Bt* HD. Comparisons (i) and (ii) were conducted to test whether the volatiles released by undamaged *Bt* or non-*Bt* rice plants were attractive to the parasitoid wasps, while comparison (iii) tested whether the wasps could distinguish between undamaged *Bt* and non-*Bt* rice plants, i.e., whether the *Bt* transformation had caused a change in the volatile emission of the rice plants. Comparisons (iv), (v), (vi), and (vii) tested whether the damage to rice plants caused by 3^rd^-instar *C. suppressalis* larvae would affect the volatile emission profile of either non-*Bt* or *Bt* rice plants and affect the host-searching behavior of the parasitoid *C. chilonis*. Comparison (viii) tested whether the parasitoid wasps could differentiate between the volatiles emitted by damaged *Bt* and non-*Bt* rice plants. In particular, the comparison between herbivore-damaged non-*Bt* rice plants and undamaged *Bt* rice plants (vii) reflected the field situation where lepidopteran larvae cause negligible or no damage to the *Bt* plants^24^. With these comparisons, two research hypotheses could be tested: i) the insertion of *Bt* genes will not change the production of rice plant volatiles; and ii) the non-*Bt* rice plants are more damaged by *C. suppressalis* than the *Bt* plants and consequently release qualitatively and quantitatively different volatile bouquets, resulting in higher attractiveness to parasitoid wasps.

### Response of *C. chilonis* to rice plants damaged by 1^st^- or 3^rd^-instar *C. suppressalis* larvae

An additional olfactometer bioassay was conducted to compare the responses of *C. chilonis* females to *Bt* rice plants that had been infested with 1^st^-instar caterpillars, which might occur in the fields, and non-*Bt* rice plants that had been infested with 1^st-^ or the 3^rd^-instar larvae of *C. suppressalis*. The material and methods used in this experiment were the same as described above. The female parasitoids were exposed to two pairs of odor sources: (i) non-*Bt* rice plants infested with 1^st^-instar *C. suppressalis* larvae vs. *Bt* rice plants infested with 1^st^-instar *C. suppressalis* larvae (1^st^ non-*Bt* HD vs. 1^st^ *Bt* HD); and (ii) non-*Bt* rice plants infested with 3^st^-instar *C. suppressalis* larvae vs. *Bt* rice plants infested with 1^st^-instar *C. suppressalis* larvae (3^rd^ non-*Bt* HD vs. 1^st^ *Bt* HD); both comparisons reflected the field situation. Each plant was infested with 30 newly hatched 1^st^-instar *C. suppressalis* larvae (<2 h) or 10 3^rd^-instar larvae. After 24 h feeding, the *C. suppressalis* damaged plants were used for the olfactometer bioassays using the method as described above.

### Collection of headspace volatiles

Volatiles were collected as described by Sun *et al.*^34^ and Zhao *et al.*^54^ with minor modifications. Volatiles were collected from both uninfested and *C. suppressalis*-infested *Bt* and non-*Bt* rice plants that were used in the bioassays. Air was filtered through activated charcoal, molecular sieves (5 Å, beads, 8–12 mesh, Sigma-Aldrich), and silica gel Rubin (cobalt-free drying agent, Sigma-Aldrich) before entering the glass bottles. Air was blown in through the base collection ports of the bottles and drawn out at the top ports of the bottles through Super Q traps (80/100 mesh, Alltech Associates, Deerfield, IL, USA). Before volatiles were collected, the system was purged for 20 min at 600 ml/min. Subsequently, the volatiles emitted from the top of the sample bottles were trapped on 100 mg Super Q in a glass tube (5 mm diameter, 8 cm height) plugged with glass wool at a flow of 400 ml/min for 8 h. The insects were left on the plants during volatile collection. For each treatment, 7 to 10 collections (from 7 to 10 replicate bottles) were made simultaneously. As a negative control, volatiles also were collected from a bottle containing an aluminum foil-wrapped flask that was filled with distilled water only. Volatile compounds that were detected in the control were considered to be air contaminants and were excluded from the plant samples.

Volatiles collected by Super Q traps were extracted with 600 μl of methylene chloride, and 2000 ng of 1-tridecene in 10 μl of methylene chloride was added to the samples as an internal standard (IS). All extracts were stored at −30 °C.

### Identification and quantification of volatiles

An Agilent gas chromatography (GC) (6890N) coupled with a mass spectrometry (MS) system (5973 MSD; Agilent Technologies, Inc., Palo Alto, CA, USA) was used for separation and detection of plant volatiles. A 1-μl volume of each sample was injected into a DB5-MS column (30 m × 0.25 mm ID × 0.25 μm film thickness; Agilent Technologies). The inlet was operated in a split-less injection mode, and the injector, ion source, and mass quadrupole were maintained at 250 °C, 230 °C, and 150 °C, respectively. Helium was used as the carrier gas with a flow of 1.0 ml min^−1^ in constant flow mode. The GC-MS was operated in scan mode with a mass range of 33–300 amu at 5.24 s^−1^ and was in an electron impact ionization mode (EI) at 70 eV. The GC oven temperature was initially kept at 40 °C for 2 min and then increased to 250 °C at 6 °C min^−1^ and kept for 2 min at 250 °C.Collected volatiles were identified by comparing their retention time and mass spectra with authentic standard compounds. If standards were unavailable, tentative identifications were made based on referenced mass-spectra available from the Nist 05 library (Scientific Instrument Services, Inc., Ringoes, NJ, USA) or previous studies^33^,^34^. The total ion chromatogram peak area of the volatile compounds was calculated by Enhanced Data Analysis software D.02.00.275 MSD Chemstation (Agilent Technologies, Inc., Palo Alto, CA, USA).

### Statistical analyses

Student’s *t*-tests were used to compare the body-weight increase of the *C. suppressalis* larvae that had fed on *Bt* or non-*Bt* rice stems. Heterogeneity among different plant pairs for each pairwise comparison was tested by the method of replicated G-tests of goodness-of-fit^55^. As different plant pairs were homogeneous in each comparison (all *P *> 0.05), data were pooled. The behavioral responses of the female wasps to pairs of odors were analyzed using Chi-square (χ^2^) tests, with an expected response of 50% for either olfactometer arm. Data for individuals that made no choice within 5 min were excluded from the analyses. The datasets of the ratios of recorded peak area of each volatile or the total volatile relative to the internal standard were log_10_(*x *+ 1) transformed before the analyses to satisfy the assumption of homogeneity of variance. Consequently they were analyzed using one-way ANOVA, and Tukey honestly significant difference (HSD) tests were conducted to separate the means between the treatments (*Bt* UD, non-*Bt* UD, *Bt* HD, and non-*Bt* HD). All statistical analysis were conducted with SPSS 22.0 (IBM SPSS, Somers, NY, USA).

## Additional Information

**How to cite this article**: Liu, Q. *et al.* *Bt* rice does not disrupt the host-searching behavior of the parasitoid *Cotesia chilonis.* *Sci. Rep.* **5**, 15295; doi: 10.1038/srep15295 (2015).

## Figures and Tables

**Table 1:** Volatiles collected from the headspace of undamaged *Bt* rice plants (*Bt* UD), undamaged non-*Bt* rice plants (non-*Bt* UD), herbivore *C. suppressalis*-damaged *Bt* rice plants (*Bt* HD), and herbivore-damaged non-*Bt* plants (non-*Bt* HD).

Peak no.	Plant volatile	Retention time (min)	Mean quantities of volatiles*			
			*Bt* UD	Non-*Bt* UD	*Bt* HD	Non-*Bt* HD
1	2-heptanone	5.14	28 ± 6 a	11 ± 4 a	331 ± 117 b	163 ±43 b
2	2-heptanol	5.34	14 ± 4 a	10 ± 3 a	232 ± 57 b	107 ± 23 b
3	unknown 1	5.84	–	–	8 ± 1 a	6 ± 1 a
4	α-piene	5.98	8 ± 2 a	10 ± 3 a	9 ± 1 a	16 ± 4 a
5	unknown 2	8.23	–	–	6 ± 1 a	5 ± 1 a
6	D-limonene	8.31	33 ± 4 a	26 ± 4 a	238 ± 30 b	204 ± 40 b
7	unknown 3	9.09	4 ± 0 a	4 ± 0 a	8 ± 1 a	6 ± 1 a
8	unknown 4	9.38	–	–	5 ± 1 a	6 ± 1 a
9	2-nonanone	9.97	–	–	89 ± 23 a	40 ± 5 a
10	linalool	10.21	163 ± 38 a	104 ± 22 a	180 ± 52 a	144 ± 36 a
11	methyl salicylate	12.41	–	–	51 ± 38 a	36 ± 18 a
12	copaene	16.44	4 ± 1a	5 ± 1 a	18± 2 b	23 ± 5 b
13	β-caryophyllene	17.41	6 ± 1 a	8 ± 2 a	11 ± 3 a	13 ± 3 a
14	α-bergamotene	17.74	–	–	5 ± 2 a	5 ± 1 a
15	germacrene D	18.67	–	–	9 ± 2 a	12 ± 2 a
16	2-tridecanone	19.07	–	–	64 ± 21 a	25 ± 7 a
17	δ-selinene	19.54	4 ± 1 a	6 ± 2 a	27 ± 5 b	31 ± 8 b
18	α-cedrene	19.70	–	–	16 ± 8 a	26 ± 6 a
	Total		251 ± 44 a	167 ± 29 a	1145 ± 256 b	805 ± 93 b

“−” indicates that the concentration of the volatile was below the detection level.

^*^Values are mean normalized quantities of volatiles (%) = 
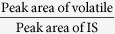
  ± SE of seven to 10 replicates. Means with the different letters in the same row are significantly different (*P *< 0.05, Tukey HSD test). Data were log_10_(*x* + 1) transformed before analysis.
